# Linagliptin treatment is associated with altered cobalamin (VitB12) homeostasis in mice and humans

**DOI:** 10.1038/s41598-023-27648-7

**Published:** 2023-01-12

**Authors:** Harald Tammen, Martin Kömhoff, Denis Delić, Søren S. Lund, Berthold Hocher, Sandra Frankenreiter, Rüdiger Hess, Maximilian von Eynatten, Michael Mark, Thomas Klein

**Affiliations:** 1 PXBioVisioN GmbH, Feodor-Lynen-Straße 31, 30625 Hannover, Germany; 2 Department of Pediatrics, University Marburg, Marburg, Germany; 3 Boehringer Ingelheim GmbH & Co. KG, Birkendorfer Str. 65, Biberach, Germany; 4 Boehringer Ingelheim International GmbH, Ingelheim, Germany; 5 Fifth Department of Medicine (Nephrology/Endocrinology/Rheumatology), University Medical Centre Mannheim, University of Heidelberg, Mannheim, Germany; 6 Key Laboratory of Study and Discovery of Small Targeted Molecules of Hunan Province, School of Medicine, Hunan Normal University, Changsha, China; 7 Reproductive and Genetic Hospital of CITIC-Xiangya, Changsha, China; 8 Institute of Medical Diagnostics, IMD Berlin, Berlin, Germany; 9 Department of Nephrology, Klinikum Rechts Der Isar, Technische Universität München, Munich, Germany

**Keywords:** Randomized controlled trials, Preclinical research, Type 2 diabetes, Chronic kidney disease

## Abstract

Linagliptin is a dipeptidyl peptidase-4 (DPP-4) inhibitor used for the treatment of type 2 diabetes, with additional beneficial effects for the kidney. Treatment of mice with linagliptin revealed increased storage of cobalamin (Cbl, Vitamin B12) in organs if a standard Cbl diet (30 µg Cbl/kg chow) is given. In order to translate these findings to humans, we determined methylmalonic acid (MMA), a surrogate marker of functional Cbl homeostasis, in human plasma and urine samples (n = 1092) from baseline and end of trial (6 months after baseline) of the previously completed MARLINA-T2D clinical trial. We found that individuals with medium Cbl levels (MMA between 50 and 270 nmol/L for plasma, 0.4 and 3.5 µmol/mmol creatinine for urine, at baseline and end of trial) exhibited higher MMA values at the end of study in placebo compared with linagliptin. Linagliptin might inhibit the N-terminal degradation of the transcobalamin receptor CD320, which is necessary for uptake of Cbl into endothelial cells. Because we demonstrate that linagliptin led to increased organ levels of Cbl in mice, sustained constant medium MMA levels in humans, and inhibited CD320 processing by DPP-4 *in-vitro*, we speculate that linagliptin promotes intra-cellular uptake of Cbl by prolonging half-life of CD320.

## Introduction

Dipeptidyl peptidase-4 (DPP-4) inhibitors (gliptins) are oral antidiabetic drugs which lead to a postprandial insulin secretion by inhibiting the degradation of glucagon-like peptide 1 (GLP-1) and glucose-dependent insulinotropic peptide^1^. DPP-4 inhibitors are also reported to have beneficial effects in experimental chronic kidney disease (CKD) animal models^2^ as well as in reducing albuminuria progression in humans^3^.

In a previous study, we investigated the effects of the DPP-4 inhibitor linagliptin in the kidneys of GLP-1 receptor knockout and wild-type mice after 5/6 nephrectomy (5/6Nx)^4^ to elucidate GLP-1 receptor independent renoprotective effects. Alongside various analytical methods, multiplex mass spectrometry (MS) was also employed. An unexpected finding in the MS data of this study was that increased cobalamin (Cbl, vitamin B12) levels were found in mice kidneys following linagliptin treatment. In the current study using MS, we analyzed the effects of varying both the dietary supplementation of Cbl as well as treatment duration of linagliptin on Cbl storage in kidneys and other organs (heart, liver, and spleen) in mice.

To determine a possible link between different Cbl levels and linagliptin treatment in humans, we analyzed methylmalonic acid (MMA) levels in human plasma and urine specimens (n = 1092 from 301 individuals) from the completed Efficacy, Safety and Modification of Albuminuria in Type 2 Diabetes Subjects with Renal Disease with LINAgliptin (MARLINA-T2D) clinical trial^5,6^. We hypothesize that linagliptin might improve Cbl storage in mice and humans by increasing the half-life of CD320, a cell surface receptor that binds transcobalamin saturated with Cbl (TC-Cbl), which possesses an N-terminal consensus DPP-4 cleavage motif in CD320 in mice and humans. To test this hypothesis, we analyzed, in vitro, whether CD320 is processed by DPP-4, i.e., an N-terminal cleavage of a dipeptide occurs and if it can be inhibited by linagliptin. Finally, we aimed to determine CD320 levels and to characterize the N-terminal portion in tissue samples using commercially available assays and an MS-based homebrew assay.

## Results

### Mass spectrometry analysis (MS) of Cbl

**Figure 1:**
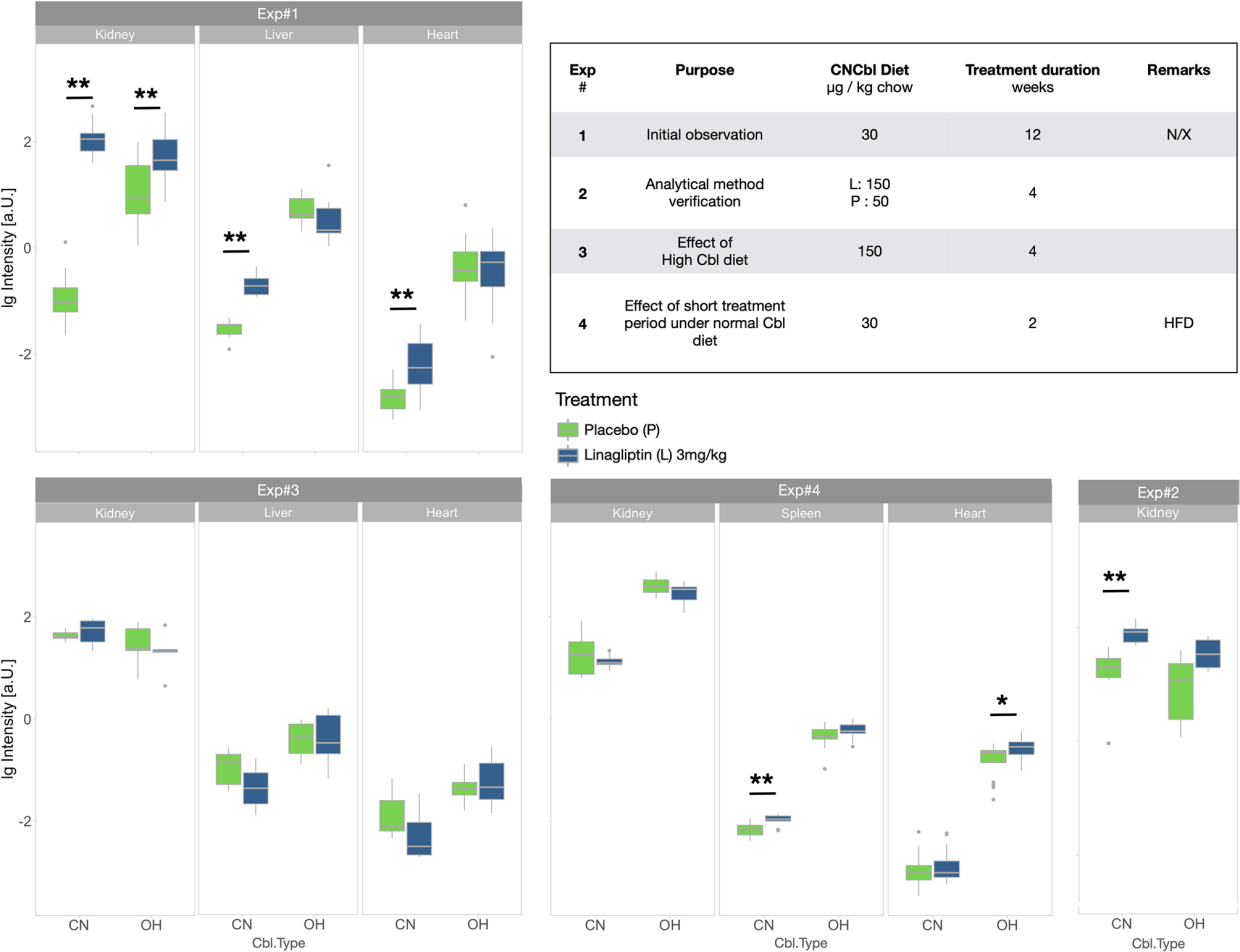
MS detection of CN and OH cobalamin in different organs from 4 independent mice studies. Box-and-whisker of log OHCbl and CNCbl standardized intensities obtained from experiments listed in Table 1. The inset shows the basic experimental parameters. The number of data points are for Exp#1:88; Exp#2: 12; Exp#3: 36; Exp#4: 96. **Significant differences between linagliptin treatment and placebo. CNCbl, cyanocobalamin; OHCbl, Hydroxycobalamin, lg, natural logarithm; HFD, high fat diet; N/X, 5/6 nephrectomy.

A set of four experiments were conducted to determine the effect of linagliptin treatment on Cbl levels in various mouse organs (Table 1 and Fig. 1) by changing the duration of linagliptin treatment and cyanocobalamin (CNCbl) dietary intake.

**Table 1:** Experimental parameters and mean values of Cbl forms in different organs.

Experiment #	All animals (n)	Linagliptin (n)	Treatment duration (Days)	Organ	Cobalamin (µg/kg chow)	Cobalamin Type	Mean Control ± SD [a.U.]	Mean Linagliptin ± SD [a.U.]	*p* TT	*p* KW
1	38	14	84	Kidney	30	OH	0.96 ± 0.51	1.74 ± 0.80	**0.0039**	**0.0020**
						CN	0.12 ± 0.06	2.37 ± 0.78	** < 0.0001**	** < 0.0001**
	10	5	84	Liver	30	OH	0.62 ± 0.19	0.60 ± 0.41	0.9230	0.2620
						CN	0.06 ± 0.01	0.15 ± 0.04	**0.0011**	**0.0040**
	38	14	84	Heart	30	OH	0.24 ± 0.15	0.22 ± 0.11	0.9230	0.9760
						CN	0.02 ± 0.00	0.04 ± 0.02	**0.0019**	**0.0003**
2	10	5	28	Kidney	Lina: 150 Controls: 50	OH	0.66 ± 0.36	1.10 ± 0.31	**0.0459**	0.1090
						CN	0.61 ± 0.26	1.17 ± 0.21	**0.0021**	**0.0040**
3	10	5	4	Kidney	150	OH	1.70 ± 0.66	1.46 ± 0.52	0.4980	0.2620
						CN	1.59 ± 0.16	1.76 ± 0.44	0.4200	0.5220
	10	5	4	Heart	150	OH	0.10 ± 0.03	0.12 ± 0.06	0.4930	0.7490
						CN	0.05 ± 0.03	0.03 ± 0.02	0.2690	0.1090
	10	5	4	Liver	150	OH	0.26 ± 0.09	0.28 ± 0.14	0.8030	0.8730
						CN	0.12 ± 0.04	0.09 ± 0.04	0.1230	0.0782
4	8	4	14	Kidney	30	OH	1.81 ± 0.30	1.57 ± 0.26	**0.0204**	0.0546
						CN	1.80 ± 0.80	1.43 ± 0.17	0.0864	0.5460
	8	4	14	Spleen	30	OH	0.10 ± 0.02	0.11 ± 0.02	0.1280	0.1870
						CN	0.05 ± 0.01	0.06 ± 0.01	** < 0.0001**	**0.0001**
	8	4	14	Heart	30	OH	0.06 ± 0.02	0.08 ± 0.01	**0.0262**	**0.0288**
						CN	0.02 ± 0.01	0.03 ± 0.01	0.5000	0.6780

*a.U.* arbitary unit; *CN* cyano; *KW* Kruskal–Wallis; *Lina* linagliptin; *OH* hydroxy; *SD* standard deviation; *TT* Welch t-test.

An overview of experiments (Exp#1–4) conducted in mice to determine the relative Cbl amount in different organs at different Vit-B12 diets and treatment durations under linagliptin and placebo. Significant *p* values (< 0.05) are in bold.

*Experiment 1*. Initial observation.

Twevle weeks of linagliptin treatment of mice under a normal Cbl diet revealed a significant increase of CNCbl compared to controls in all organs (kidney, liver, heart) analyzed.

*Experiment 2*. Verification of the method for semi-quantification of Cbl by MS.

Since we used a semi-quantitative method for Cbl determination, we designed an experiment to demonstrate the capability of the MS-assay. Therefore, animals were treated with either linagliptin for 4w and received a high Cbl diet (150 µg/kg chow) or placebo and a Cbl diet of 50 µg/kg chow. A significant increase of CNCbl in kidneys could be observed (as expected) in animals receiving the higher CNCbl diet.

*Experiment 3*. High Cbl-diet.

Animals were fed with a high Cbl diet (150 µg/kg chow) for 2 weeks and were treated with linagliptin or placebo for 4 days. Placebo and linagliptin-treated animals showed no significant differences in Cbl levels between both groups in any organ analyzed.

*Experiment 4*. Normal Cbl diet.

Animals were fed a standard CNCbl (but high fat diet) and treated with linagliptin for 2 weeks. A significant increase of CNCbl in spleen and hydroxycobalamin (OHCbl) in heart tissue samples could be observed.

### MMA ELISA

To assess effects of linagliptin on Cbl homeostasis in humans, MMA levels were measured in urine (uMMA) and plasma (pMMA) samples from the MARLINA-T2D clinical trial. The rational to measure these surrogate markers of Cbl homeostasis is that pMMA and uMMA levels are increased if the vitamin-B12-dependent methylmalonyl-CoA mutase lacks sufficient Cbl to convert L-methylmalonyl-CoA to succinyl-CoA.

**Figure 2:**
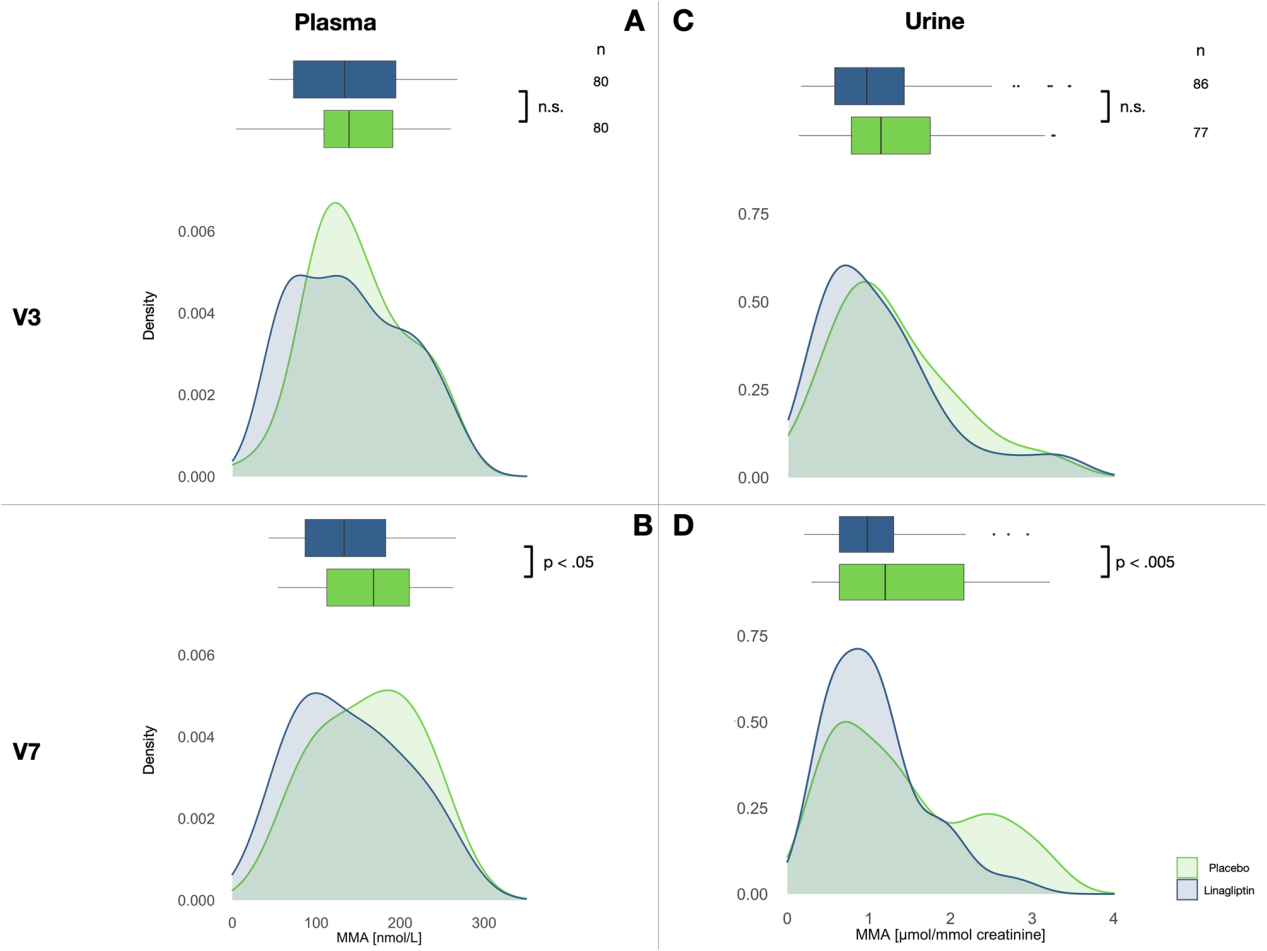
Distribution of MMA values in plasma and urine at V3 and V7 in individuals with medium MMA levels in dependence of treatment. MMA values for plasma (nmol/L, left, A and B) and urine samples (µmol/mmol creatine, right, C and D) for placebo (green) and linagliptin (blue) at V3 (top, A and C), and V7 (bottom, B and D). The number of samples in each group is shown at the top right. Statistically significant differences between placebo and linagliptin are indicated (Welch t-test). Data is shown as a box-and-whisker plot and the corresponding kernel density estimation. The data shows a shift of MMA values at V7 to the right (towards higher MMA values) in the Placebo group. The shift is also depicted in median and mean values shown in Tables 5 and 6, respectively. MMA, methylmalonic acid; n.s., not significant; V3, baseline; V7, end of study.

Samples (n = 1092 from 301 individuals) from MARLINA-T2D (360 participants) at baseline (V3) and at end of the trial (V7: baseline [V3] + 6 months) were analyzed to determine pMMA and uMMA levels in a subset of the study cohort (Tables 2 and 3). Overall, no significant differences in MMA levels between placebo and linagliptin were observed, but significant differences were seen in different ethnic groups (Table 3). In individuals of Asian descent, significantly higher pMMA (*p* < 0.01, Cohen’s d estimate 0.99 [95% CI 0.77–1.20]) and uMMA levels (*p* < 0.01, Cohen’s d estimate 0.38 [95% CI 0.17–0.58]) were found compared with White patients, unrelated to linagliptin treatment. MMA levels are primarily determined by Cbl homeostasis, i.e., linagliptin alone is unlikely to improve the Cbl status if, for example, the Cbl supply is insufficient. Therefore, linagliptin treatment does not necessarily result in lower MMA levels in patients. In contrast to the animal model, Cbl supply varies in the human study population, which is one crucial determinant of MMA levels. Consequently, analysis of MMA was re-evaluated in dependence of the MMA levels at V3 and V7. Therefore, the collective was grouped into low, medium and high MMA levels. Individuals with mean MMA values at V3 and V7 below 50 nmol/L in plasma and 0.4 µmol/mmol creatinine in urine were classified as low, individuals with MMA values below the upper clinical reference value (270 nmol/L for plasma and 3.5 µmol/mmol creatinine for urine) at V3 and V7 but above the lower threshold as medium, and the remainder as high. High MMA levels have a higher probability to indicate individuals who had an insufficient Cbl supply during the study duration, whereas medium MMA levels have a higher probability to indicate individuals who had an adequate Cbl supply during the study duration, and whereas low MMA levels have a higher probability to indicate individuals with an ample Cbl supply during the study duration. Based on the animal experiments, we speculated that effects of linagliptin would be most likely detectable in the medium group. By applying these thresholds, 53% (n = 160 individuals) of plasma samples and 72% (n = 163 individuals) of urine samples were assigned to medium MMA levels (Table 4). Comparison of medium MMA values from V7, but not V3, between placebo and linagliptin revealed significantly higher levels both for uMMA (*p* < 0.005, Cohen’s d estimate 0.48 [95% CI 0.15–0.81) and pMMA (*p* < 0.05, Cohen’s d estimate 0.34 [95% CI 0.04–0.69]) in placebo (Fig. 2, Tables 4, 5, 6). The distribution (density estimation) of pMMA and uMMA values demonstrates a right shift in the placebo group whereas it remained constant in the Linagliptin group suggesting an altered Cbl homeostasis. A statistical analysis, conducted at different MMA threshold levels between placebo and linagliptin is shown in Supplementary Fig. S1a and b supporting the observation of a significant effect at medium MMA levels at V7 in both urine and plasma.

**Table 2:** Patient characteristics and pMMA and uMMA values of a subset of individuals from the MARLINA trial. Number of samples in each group and subgroup.

Groups	Plasma	Urine
Total	301	245
White	60	60
Asian	183	183
Other/Unknown	58	2
Linagliptin	151	120
Placebo	150	125

**Table 3:** Patient characteristics and pMMA and uMMA values of a subset of individuals from the MARLINA trial. Arithmetic means and standard deviations of MMA values in plasma and urine per point in time and group.

MMA Levels point in time	Plasma [nmol/L]		Urine [µmol/mmol Creatinine]	
	V3	V7	V3	V7
All	222 ± 131	248 ± 149	2.05 ± 2.78	2.14 ± 3.17
White	152 ± 92	154 ± 102	1.4 ± 2.3	1.34 ± 2.59
Asian*	248 ± 139	278 ± 150	2.3 ± 2.9	2.42 ± 3.31
Linagliptin	225 ± 145	246 ± 158	1.77 ± 2.23	1.99 ± 3.02
Placebo	220 ± 117	250 ± 140	2.34 ± 3.2	2.29 ± 3.31

*MMA* methylmalonic acid; *V3* baseline; *V7* end of study.

*Significantly higher plasma and urine MMA levels in Asian patients compared with White patients (Welch -test).

**Table 4:** Patient characteristics and pMMA and uMMA values of a subset of individuals from the MARLINA trial. Number of individuals with low, medium and high pMMA and uMMA levels grouped by Race and Sex.

	Group	Race	Sex	n	pMMA nmol/L			uMMA µmol/mmol Creatinine		
					Low < 50	Medium 50–270	High ≥ 270	Low < 0.4	Medium 0.4–3.5	High ≥ 3.5
1	Placebo	Asian	Female	36	0	14	22	2	20	14
2		Asian	Male	59	0	31	28	5	41	13
3		Other/unknown	Male	1	0	1	0	1	0	0
4		Unknown	NA	25	0	13	12			
5		White	Female	7	0	7	0	3	3	1
6		White	Male	22	2	14	6	5	13	4
7	Linagliptin 5 mg	Asian	Female	34	0	18	16	2	23	9
8		Asian	Male	54	0	20	34	5	38	11
9		Other/unknown	Female	1	0	0	1	0	1	0
10		Unknown	NA	31	0	17	14			
11		White	Female	11	0	9	2	1	8	2
12		White	Male	20	0	16	4	3	16	1

*NA* not applicable; *pMMA* plasma methylmalonic acid; *uMMA* urine methylmalonic acid.

**Table 5:** Patient characteristics and pMMA and uMMA values of a subset of individuals from the MARLINA trial. Median and IQR of pMMA and uMMA at V3 and V7 group by treatment group and MMA levels.

MMA levels	Group	n	Median V3	IQR V3	Median V7	IQR V7
**pMMA** **nmol/L**						
High	Placebo	68	300	116	330	126
High	Linagliptin 5 mg	71	302	155	347	128
Mid	Placebo	80	138	82.5	169	98.7
Mid	Linagliptin 5 mg	80	133	123	134	96.5
**uMMA** **µmol/mmol Creatinine**						
High	Placebo	32	4.150	6.780	3.730	3.830
High	Linagliptin 5 mg	23	3.850	2.760	4.870	3.540
Mid	Placebo	77	1.140	0.972	1.200	1.530
Mid	Linagliptin 5 mg	86	0.968	0.857	0.978	0.666
Low	Placebo	16	0.324	0.198	0.287	0.131
Low	Linagliptin 5 mg	11	0.292	0.0998	0.235	0.167

*IQR* interquartile range; *pMMA* plasma methylmalonic acid; *uMMA* urine methylmalonic acid; *V3* baseline; *V7* end of study.

**Table 6:** Patient characteristics and pMMA and uMMA values of a subset of individuals from the MARLINA trial. Mean and SD of MMA, eGFR, Age and BMI at V3 and V7 at medium MMA levels.

Time	Variable	Placebo		Linagliptin		TT
		n	Mean ± SD	n	Mean ± SD	*p*
**Plasma**						
V3	MMA	80	149.0 ± 57.1	80	139.0 ± 65.2	0.300
	eGFR		78.9 ± 22.8		77.9 ± 22.4	0.700
	Age		60.4 ± 9.2		62.4 ± 10.3	0.200
	BMI		28.8 ± 4.7		28.3 ± 4.8	0.500
V7	MMA		162.0 ± 58.9		141.0 ± 64.8	**0.030**
	eGFR		77.6 ± 24.1		75.8 ± 23.0	0.600
	BMI		28.9 ± 4.9		28.3 ± 4.8	0.500
**Urine**						
V3	MMA	77	1.3 ± 0.75	86	1.14 ± 0.77	0.200
	eGFR		78.6 ± 21.9		81.6 ± 19.8	0.300
	Age		59.1 ± 9.6		60.7 ± 8.9	0.300
	BMI		27.6 ± 4.1		27.7 ± 4.7	0.900
V7	MMA		1.42 ± 0.87		1.06 ± 0.58	**0.003**
	eGFR		76.7 ± 22.9		79.0 ± 20.8	0.500
	BMI		27.4 ± 4.2		27.7 ± 4.7	0.700

*eGFR* estimated glomerular filtration; *MMA* methylmalonic acid; *SD* standard deviation; *TT* Welch t-test; *V3* baseline; *V7* end of study.

Significant *p* values (< 0.05) are in bold. BMI, body mass index.

### CD320 cleavage

**Figure 3:**
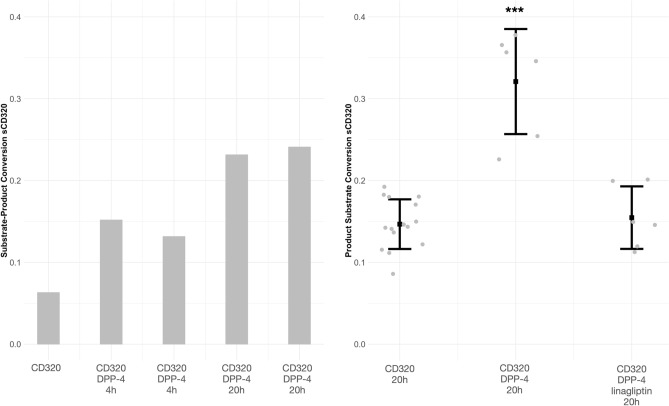
N-terminal processing of CD320. Substrate-product ratios of tryptic peptides derived from the N-terminal part of CD320 (CD320_36-58: SPLSTPTSAQAAGPSSGSCPPTK and CD320_38-58: LSTPTSAQAAGPSSGSCPPTK). The conversion rate in each group is given as mean + /−  SD and individual data points. Left: The column bars show a time-dependent increase in conversion rate due to DPP-4 incubation time. Right: The ratio between both tryptic peptides is depicted as increase in conversion rate due to DPP-4 and inhibition of increase by linagliptin (***p < 0.0005). DPP-4, dipeptidyl peptidase-4; sCD320, soluble form of CD320.

To determine if CD320 is a substrate of the proteolytic activity of DPP-4, cleavage experiments were conducted using mass spectrometric measurements of surrogate peptides. The analysis revealed a time-dependent increased conversion rate in presence of DPP-4 (*p* < 0.0005, Cohen’s d estimate 3.37 [95% CI 2.1–4.6]) without linagliptin (Fig. 3, Supplementary Fig. S2), indicating processing of CD320 by DPP-4.

To verify the hypothesis of increased CD320 in organs due to linagliptin treatment, we measured CD320 levels using a commercially available ELISA and evaluated the use of antibodies for capturing CD320 from tissue extracts. Surprisingly, CD320 protein could not be detected. Next, we evaluated two antibodies (AB1 and AB2) to capture the CD320 protein. AB1 was not able to capture the protein whereas AB2 recognized the HIS-tag of the expressed protein only (Supplementary Fig. S3). Although processing was observed in vitro, ultimately, we were unable to determine CD320 levels and its processing in the tissue samples.

## Discussion

Cbl subsumes several chemical forms of vitamin B12 and is classified by its axial ligand of the cobalt ion. Dietary B12 is often taken up as CNCbl or OHCbl and is converted by humans and animals into active Cbl compounds involving removal of the upper-axial ligand yielding the one-electron reduced intermediate cob(II)alamin for Cbl dependent enzymes. The dietary uptake, transport, and cellular influx of Cbl requires a complex process involving multiple proteins and receptors (Supplementary Fig. S4)^7^. In an animal study (Exp#1, Table 1), a significant accumulation of CNCbl in murine kidney (20-fold), liver (twofold) and heart (twofold) following linagliptin treatment over 12 weeks combined with a normal Cbl diet was observed. Shortening the duration of linagliptin treatment to 4 days and increasing the dietary amount of Cbl in a subsequent animal experiment (Exp#3, Table 1) attenuated the effect of linagliptin on Cbl accumulation, whereas in animals with a normal Cbl diet (Exp#4), an approximate 20% increase of intracellular Cbl levels (spleen and heart) following short-term (2 weeks) linagliptin treatment was seen. It is worth noting that all animals were normoglycemic apart from the animals in Exp#4.

To determine if the altered Cbl homeostasis with linagliptin seen in mice is also present in humans, MMA levels in samples from MARLINA-T2D were determined. We used MMA as a surrogate marker for Cbl homeostasis, as homocysteine, another marker used to determine Cbl homeostasis, could not be measured in plasma samples due its pre-analytical requirements for blood sampling. MMA is a sensitive but not an entirely specific marker. MMA levels may be influenced by renal function, age and a variety of other factors (e.g., microbiota).

Because, both high as well as low Cbl-diet abrogated the effect of linagliptin on tissue Cbl accumulation in mice, MMA in samples from individuals in the MARLINA-T2D clinical trial were grouped into low, medium and high levels. In line with our animal data, analysis of the MARLINA-T2D data suggests that linagliptin can affect medium MMA levels: Placebo-treated individuals possess significantly higher MMA values in plasma and urine at V7 compared with linagliptin-treated individuals at V7, but not at V3. The Cohens’d effect size is greater for uMMA than for pMMA, which might be related to better diagnostic sensitivity of uMMA in detecting Cbl deficiency in patients with diabetes^8^. It is important to note that these effects observed in patients from MARLINA-T2D are modest; however, given that the observation period (six months) is relatively short, it is possible that incremental beneficial effect of linagliptin may be apparent over a longer treatment period. Furthermore, the clinical study population of interest was limited due to selection of appropriate samples based on estimated Cbl supply.

We hypothesized that the effects of linagliptin on Cbl homeostasis in man and mice result from inhibition of DPP-4 dependent processing of major proteins involved in the Cbl pathway (Cubilin, Protein aminoless, Intrinsic factor, Haptocorrin, Megalin, Transcobalamin-2, MRP1 and CD320). The N-terminus of the CD320 protein contains a prototypical DPP-4 cleavage motif^9^ both in humans (S36P37) and mice (A29P30). All other major proteins of the Cbl pathway are devoid of this motif. CD320‚ is a cell-surface receptor for transcobalamin saturated with cobalamin (TCbl) and plays an important role in cobalamin uptake. Loss of function mutations result in functional B12 deficiency^10–12^. To determine if CD320 is a potential substrate for DPP-4, we performed in-vitro cleavage experiments of CD320. Incubation of CD320 in the presence of DPP-4 significantly increased the N-terminal dipeptide truncation and was inhibited by linagliptin, which strongly suggests that CD320 is indeed a substrate of DPP-4. DPP-4 is expressed on the plasma membrane but also exists in a soluble form^13^. It is conceivable that DPP-4 is capable of processing CD320 on the plasma membrane. A previous study^4^ demonstrated the activity of DPP-4, its inhibition by linagliptin treatment in kidney tissue homogenates and the N-Terminal processing of proteins/peptides in tissue samples.. Additionally, we have recently shown inhibition of in situ activity of DPP-4 in rat kidney by linagliptin^14^.

We aimed to determine CD320 in tissue samples by using a commercially available ELISA and antibody capturing. However, both approaches were unsuccessful due to analytical challenges (Supp. Fig. S3).

CD320 expression appears to be tightly regulated according to the proliferative and differentiation status of the cell. Of note, mRNA analyses (of heart and kidney tissues from Exp#1, Table 1) did not reveal any significant differences in CD320 expression between control and linagliptin-treated animals (data not shown). In a recently published paper^15^, the authors reported that the binding of TC-Cbl to CD320 initiates internalization of the receptor complex (routed to the endolysosomal network), CD320 and transcobalamin is then proteolytically degraded, and Cbl is finally exported into the cytosolic compartment. Therefore, the expression of receptors on the cell surface is dependent on the synthesis of new receptors. The authors induced internalization by incubating cells in medium with excess of TC-Cbl. This is in accordance with the observations we obtained i.e., adding significant amounts of Cbl to the diet resulted in non-significant differences in intracellular Cbl levels between controls and linagliptin-treated animals because of the supposedly decreased CD320 density^16^ thus reducing the potential DPP-4 processing thereby mitigating any linagliptin effect.

In summary, we demonstrate that linagliptin increased organ levels of Cbl in mice and resulted in sustained constant medium MMA levels in humans and speculate that these effects of linagliptin result from inhibition of the proteolytic cleavage of CD320 via its N-terminal DPP-4 cleavage site. However, we failed to demonstrate the processing of CD320 in tissue samples and did perform a post-hoc MMA analysis in already collected samples impeding the analysis of homocysteine due to its pre-analytical requirements. Since we analyzed a limited set of organs, we cannot exclude the possibility of decreased uptake of Cbl in other organs (e.g., brain or bone marrow). Nevertheless, we did conduct four independent animal experiments using semi-quantitative mass spectrometry to determine CNCbl and OHCBl in 150 tissue samples and could translate the findings to humans using MMA in a moderate sample size (n > 160 subjects) in two different specimens (plasma and urine) at two points in time thus increasing confidence in the conclusion drawn.

## Material and methods

### Ethics statement

All animal experiments were performed with permission of the local authorities and conducted in accordance with the German legislation and the guidelines from Directive 2010/63/EU of the European Parliament on the protection of animals used for scientific purposes and were conducted in accordance with the Practice Guidelines for Laboratory animals. The study was carried out in compliance of the ARRIVE guidelines. The experiment was carried out under supervision of an experienced and certified person at Boehringer Ingelheim, and the protocol was approved by the Boehringer Ingelheim animal welfare committee. The MARLINA-T2D protocol was approved by independent ethics committees and institutional review boards at each participating center, and the study was conducted according to the principles of the Declaration of Helsinki and the ICH Harmonised Tripartite Guidelines for Good Clinical Practice. All individuals provided written informed consent prior to participation.

### Animals and study design

*Experiment #1*. Animals were maintained under normal chow with standard 30 µg/kg CNCbl concentration for three months. Linagliptin concentration was 83 mg linagliptin/kg chow. Animals have been 5/6 nephrectomized to induced renal failure. Kidney, heart and liver were analyzed after three months for VitB12 content. The study is described in detail in a previous publication^4^.

*Experiment #2*. Male C57BL6/J mice (age 10–11 weeks) obtained from Janvier Labs (Le Genest-Saint-Isle, France) were fed a 150 µg CNCbl/kg diet (n = 5) supplemented with 83 mg/kg linagliptin (n = 5) for 4 weeks. Control animals (n = 5) during the same period were given a diet containing 50 µg CNCbl/kg chow without linagliptin. At the end of the study, the kidney was analyzed for Vit-B12 content.

*Experiment#3*. Male mice from Janvier (10–11 weeks) were fed 2 weeks and 4 days a diet containing 150 µg CNCbl/kg chow (control animals; n = 5); and linagliptin treated animal (n = 5) were fed the same diet of 150 µg CNCbl/kg for 2 weeks and subsequently treated with for 4 days with 3 mg/kg/d linagliptin. At the end of the study kidney, heart, and liver were analyzed for Vit-B12 content.

*Experiment #4*. Mice from Janvier arrived at 5 weeks of age and were made diet-induced obese (DIO) by offering ad libitum access to tap water and high-fat diet (5.15 kcal/g; 60 kcal-% fat, 20 kcal-% carbohydrate, 20% kcal-% protein) for 26–27 weeks before study start. The CNCbl content was 30 µg/kg chow. Mice (n = 4 per group) received either vehicle or linagliptin (3 mg/kg/d) for the following 14 days. Kidney, heart, and spleen were analyzed for Vit-B12 content. The study is described in detail recently^17^. Material from this study was taken in order to translate the impaired metabolic situation from patients also to mice.

### Liquid chromatography and mass spectrometry

In matrix-assisted laser desorption/ionization time-of-flight (MALDI-TOF)-MS, the upper axial ligand of the cobalt ion (e.g. –CN) of Cbl is always lost due to the applied ionization energies and replaced by a hydrogen ion, resulting in identical molecular masses for the different forms of Cbl. Since various chemical forms of Cbl possess different hydrophobicities (–OH < –CN < –Adenosyl < –CH3), separation via reversed-phased chromatography allows for an assignment of Cbl types based on elution profile, molecular mass and MS–MS fragmentation.

This method has been previously described elsewhere^18^. Briefly, low molecular weight entities were extracted from tissues, separated by reversed phase liquid chromatography and an aliquot of each lyophilized fraction was re-suspended in 15 µl of a matrix solution containing alpha- cyano-4-hydroxy cinnamic acid (HCCA, 8 mg/ml, Fluka) with 6-desoxy-L-galactose additive in 50/49/1 (v/v/v) 0.2% TFA/acetonitrile/acetone. 0.5 μl of the sample/matrix mixture were spotted on a target and subjected to MALDI-TOF-MS. After MS data acquisition, spectra were analyzed^19^. To semi-quantify CNCbl and OHCbl, 8 fmol of angiotensin or bradykinin was added prior to MS-measurement and resulting mass spectra were standardized to the intensity of angiotensin (CN) or bardykinin (OH). Two different standards for standardization for each Cbl type were used because of the background in different chromatographic fractions.

Exp#1–Exp#3 were measured using a 4700 MALDI-TOF-TOF (Applied Biosystems 4700 Proteomics Analyzer) in linear mode, whereas Exp#4 was measured in quadruplicate using a 5800 MALDI-TOF-TOF (Applied Biosystems 5800 Proteomics Analyzer) in reflectron mode.

Exp#2 served as a verification experiment to demonstrate that elevated Cbl levels can be detected in tissue samples using MS. Therefore, linagliptin treated animals were fed a higher amount of Cbl (150 µg/kg chow) in comparison to controls (50 µg /kg chow). The resulting data (Fig. 1, Table 1) shows a significant accumulation of CNCbl in the kidneys thus strongly suggesting that the MS assay is capable to relatively quantify Cbl.

For mass calibration in all measurements, a mixture of standard peptides in a mass range from 1 to 6 kDa were used. The calibration spots were homogeneously distributed over the target plate. All measurements were calibrated using a default calibration, which was updated directly prior to the sample analysis using the automatic plate calibration function of the Explorer software package (Applied Biosystems, V. 2.0). For MS/MS experiments, fragmentation was carried out in the collision-induced dissociation (CID) mode of the MALDI-TOF/TOF mass spectrometer with collision energy of 1 keV and ambient air as the collision gas at a typical pressure of 4*10–7 torr. MS/MS spectra were subsequently noise filtered and peak de-isotoped. The chromatograms and exemplary MS–MS fragment spectra of the four Cbl variants are given in Supplemental Fig. S5.

### MMA assay

Human plasma from the MARLINA trial was collected via venous puncture in EDTA-coated tubes and plasma was separated by centrifugation (2000 × *g* for 10 min at 4 °C). Urine was centrifuged (2000 × *g* for 10 min at 4 °C) and aliquots were immediately stored at − 80 °C. The assays were conducted mid-2017 for urine and early 2018 for plasma. A competitive ELISA kit (Catalog No: E2091Ge, Wuhan Eiaab Science Co., Ltd., Guangguguoji, China) was used according to the instructions by the manufacturer to determine MMA levels in urine and plasma. Briefly, a dilution series prepared from the supplied MMA standard was used to calibrate the assay. The immunoassay kit was developed for research use only and allows for the in vitro quantitative determination of MMA concentrations in serum, plasma, tissue homogenates, cell culture supernatants, and other biological fluids.

### CD320 cleavage by DPP-4

For analysis of CD320 cleavage by DPP-4, 50 pmol of purchased (antibodies-online, Aachen, Germany, ABIN2008678) recombinant CD320 was incubated for 0 h, 5 h and 20 h in HEPES buffer at pH 7.3 at 37 °C with or without 100 pmol linagliptin and/or 20 ng DPP-4. Subsequently samples were subjected to tryptic digestion and carbamidomethylation^20^, as direct determinations of the N-terminal truncated forms are challenging. Resulting digests were analyzed using reverse-phase chromatography and mass spectrometry. Peptides were identified using MS–MS fragmentation. In total, 14 independent incubations experiments (CD320: 6, CD320 + DPP-4: 6, CD320 + DPP-4 + linagliptin: 2) were performed and measured in MS in triplicate. The digestion of CD320 yields, among others, a tryptic peptide (SPLSTPTSAQAAGPSSGSCPPTK) representing the N-terminus of CD320 (CD320AA36-58). Prior incubation of CD320 with DPP-4 results in an increase of the truncated form (CD320AA38-58) of the said peptide i.e., a surrogate marker for DPP-4 cleavage. For analysis, the intensity-ratios between CD320AA38-58 and the sum of CD320AA36-58 and CD320AA38-58 were calculated to estimate the conversion rate.

### CD320 ELISA and CD320 antibody capturing

For measurement of CD320 (aa 1-208) a commercially available ELISA kit was used (ELISA kit, BG Bluegene (China), Product Code: E03C1485).

For antibody capturing of CD320 in mice tissue samples, two antibodies were purchased and evaluated. AB1 was a CD320 monoclonal antibody (M04), clone 4F2, monoclonal antibody raised against a full-length recombinant CD320, AAH00668, 1-282, Isotype: IgG2a Kappa. AB2 was produced in rabbits immunized with purified, recombinant mouse CD320 (1-208, expressed with a C-terminal polyhistidine tag, ABIN2008679 NP_062294.3). For coupling of antibodies to beaded agarose resin, the MicroLink Protein Coupling Kit (Thermo fisher Scientific, product number: 20475) was used.

### Data analysis

For data analysis, R (A Language and Environment for Statistical Computing: www.r-project.org) version 4.1.0, including packages tidyverse, rstatix, effsize, MALDIquant and reshape2 was used. For statistical testing Kruskal–Wallis rank sum test, Wilcoxon signed rank test with continuity correction, one-way ANOVA and Welch t-test were employed. For calculation of effect sizes, Cohen’s d estimate was used. Confidence intervals were calculated by bootstrap. Kernel density estimates were calculated using ggplot2.

## Supplementary Information

Supplementary Information.

## Data Availability

To ensure independent interpretation of clinical study results and enable authors to fulfill their role and obligations under the ICMJE criteria, Boehringer Ingelheim grants all external authors access to relevant clinical study data pertinent to the development of the publication. In adherence with the Boehringer Ingelheim Policy on Transparency and Publication of Clinical Study Data, scientific and medical researchers can request access to clinical study data when it becomes available on https://vivli.org/ and earliest after publication of the primary manuscript in a peer-reviewed journal, regulatory activities are complete and other criteria are met. Please visit https://www.mystudywindow.com/msw/datasharing for further information.
