# Supplementary material for: Linagliptin treatment is associated with altered cobalamin (VitB12) homeostasis in mice and humans
Source: Sci Rep. 2023 Jan 12;13:601. doi: 10.1038/s41598-023-27648-7 (PMC9837112; doi:10.1038/s41598-023-27648-7)
Supplement: Supplementary file 1 — Supplementary Information. [file 41598_2023_27648_MOESM1_ESM.docx]

**Supplementary Material**

**Supplementary Figure S1a:**

**
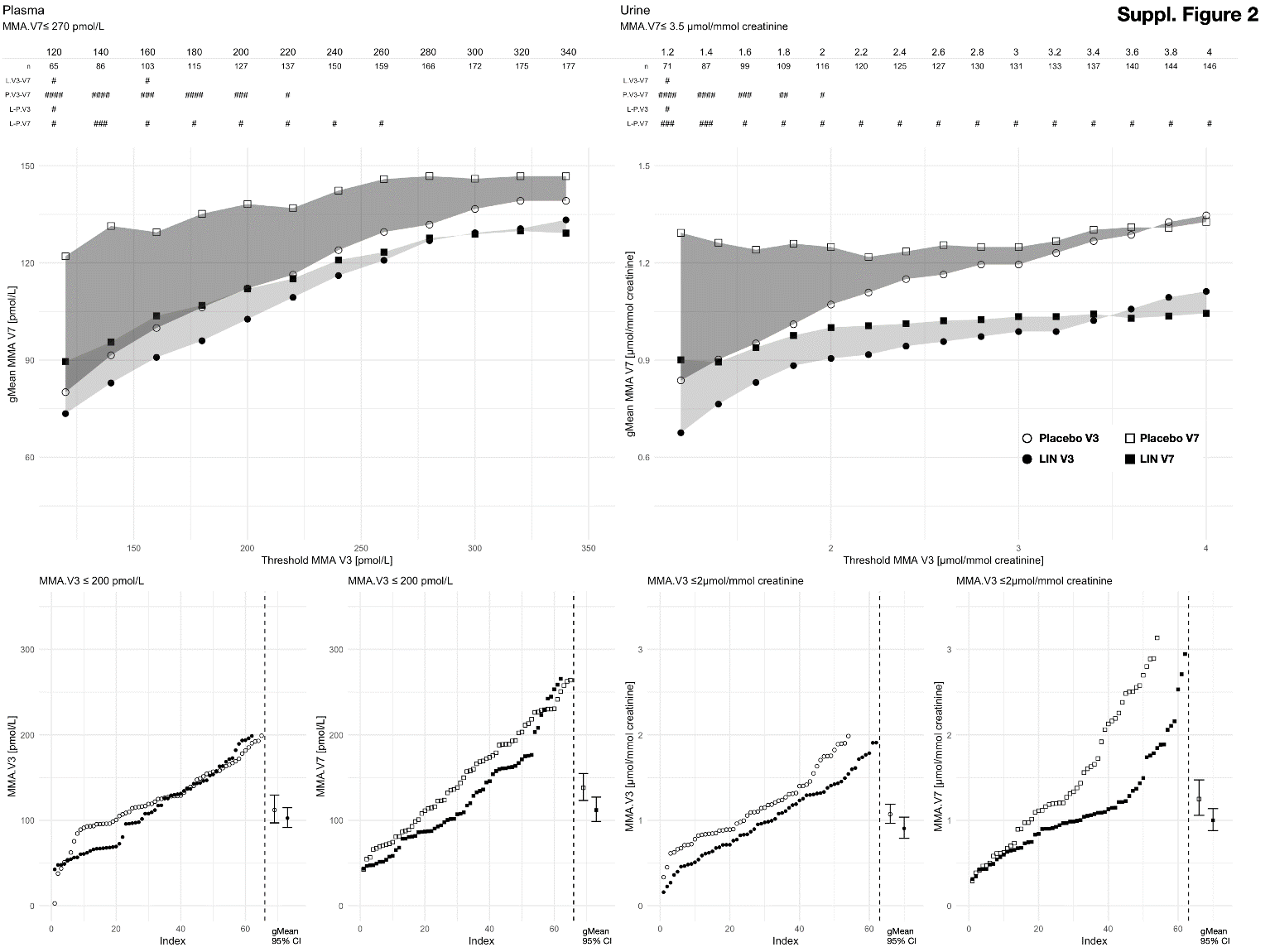
**

A statistical analysis to determine MMA levels at different MMA threshold levels.

Top: The table indicates significant differences (^#^: 0.05, ^##^: 0.01, ^###^ :0.005 ^####^: 0.001) between groups (L/P; Kruskal-Wallis test) at V3 and V7 and intra-individual significant differences per group (L.V3-V7/P.V3-V7; paired Wilcoxon test) between V3 and V7. Middle: Geometrical mean (gMean, centre) of pMMA (left) and uMMA (right) at V7 from individuals with MMA values below the upper reference value at V7 at different MMA threshold levels at V3. The plot shows a divergence between MMA values at V3 and V7 for placebo, whereas for linagliptin nearly identical curves are present. Bottom: MMA threshold levels of 200 pmol/L and 2µmol/mmol creatinine for each group at each time point in ascending order and the corresponding geometrical mean and 95% confidence interval.

CI, confidence interval; L, linagliptin; MMA, methylmalonic acid; P, placebo; V3, baseline; V7, end of study.

**Supplementary Figure S1b:**


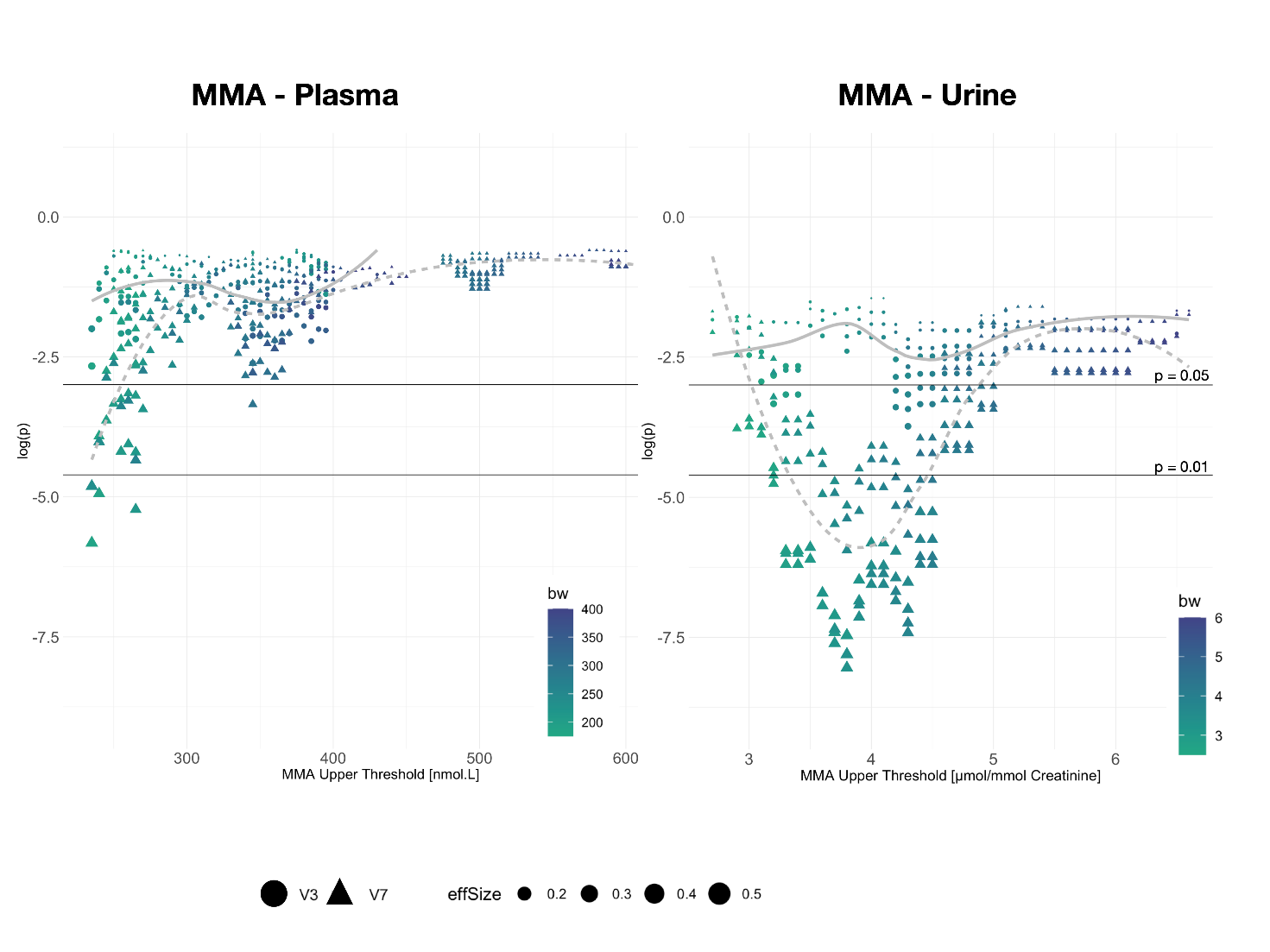


An analysis to determine significant MMA differences between Placebo and Linagliptin at V3 and V7 at different MMA threshold levels and bandwidths

The plot shows the relation between the log(p) (Welch t-test) across different MMA threshold levels at different bandwidths (span between lower threshold level and upper threshold level). Each datapoint shows the comparison between groups (Linagliptin vs. Placebo) at V3 (circle) and V7 (square) in individuals possessing the corresponding MMA level. Each data point consists of at least 130 individuals and an effect size (Cohens’d) > |0.1|. Significant p-values are below the corresponding horizontal lines. The gray lines indicate trendlines for V3 (solid) and V7 (dashed) using LOESS smooth (smoothed conditional means) with default parameters. The plot shows a high number of significant differences at V7 between Placebo and Linagliptin at medium MMA levels. The effect attenuates at high MMA levels and is more pronounced for uMMA.

bw: bandwidth, effSize: effect size.

**Supplementary Figure S2:**

**
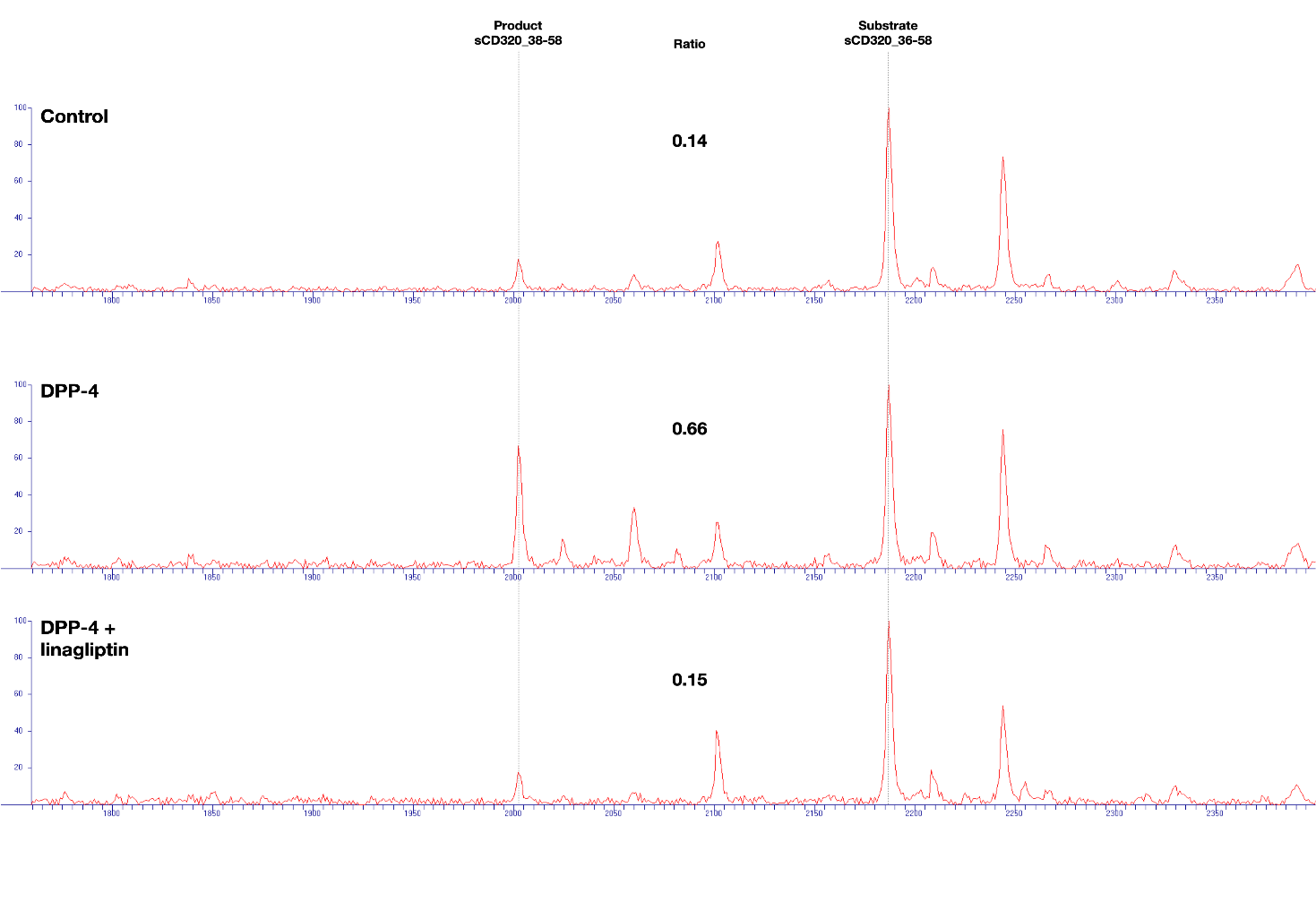
**

Exemplary mass spectra of surrogate peptides after tryptic digestion of CD320 after DPP-4 incubation in presence or absence of linagliptin. The ratio between substrate and product is indicated.

DPP-4, dipeptidyl peptidase-4; sCD320, soluble form of CD320.

**Supplementary Figure S3:**

**
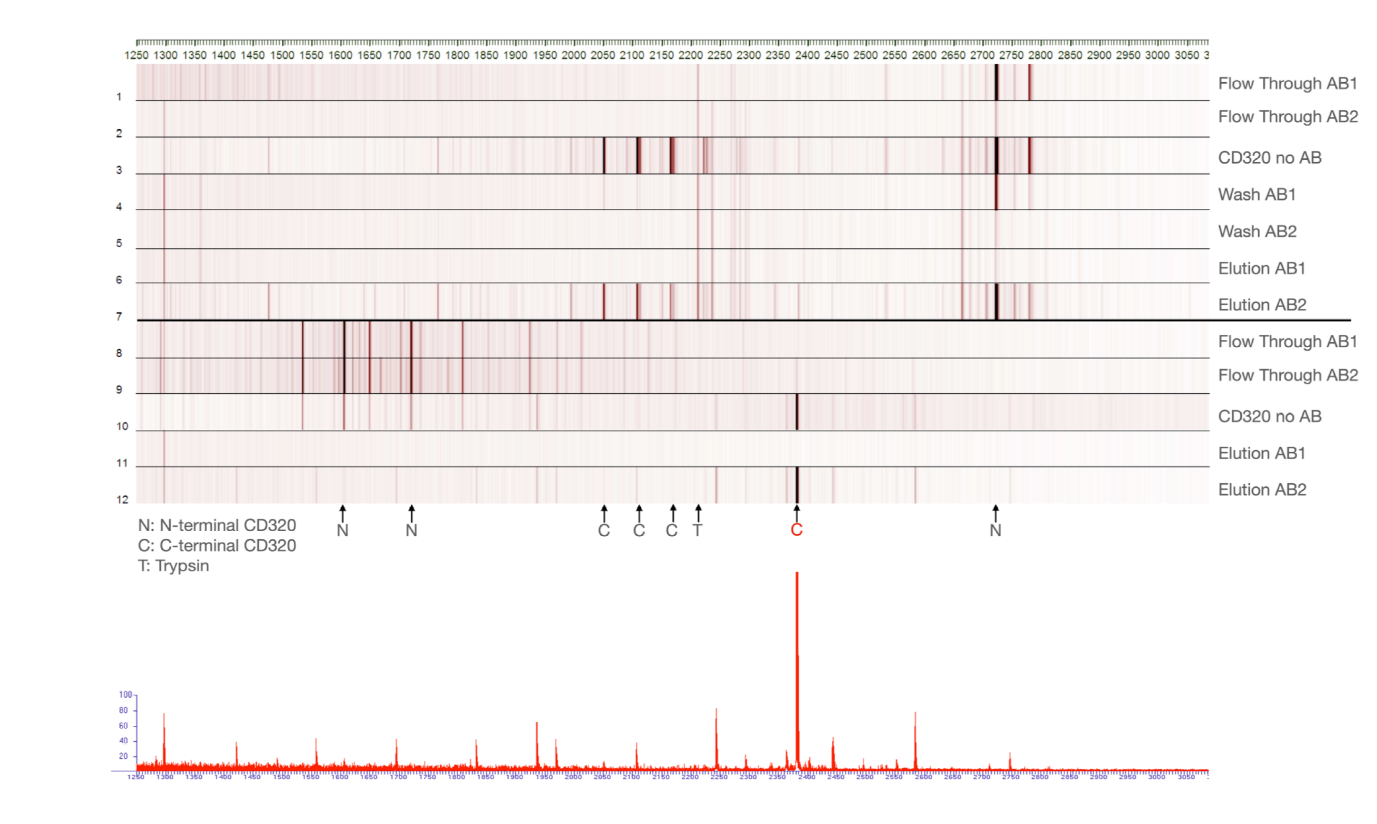
**

Antibody capturing experiments. Top: mass spectrometric data after capturing of intact CD320 and subsequent tryptic digestion (Lane 1-7) or direct measuring after capturing of peptides generated from CD320 by heat-assisted acid degradation (Lane 8-12). Lane 3 and 10 serve as controls. It is apparent that AB1 did not capture CD320 in both experiments. In contrast, AB2 was capable to capture CD320 as well as the C-terminal portion of CD320 (red C). The bottom spectrum shows the data from Lane 12. This indicates that AB2 needs the HEXAHIS Tag for epitope recognition.

AB, antibody.

**Supplementary Figure S4**

**
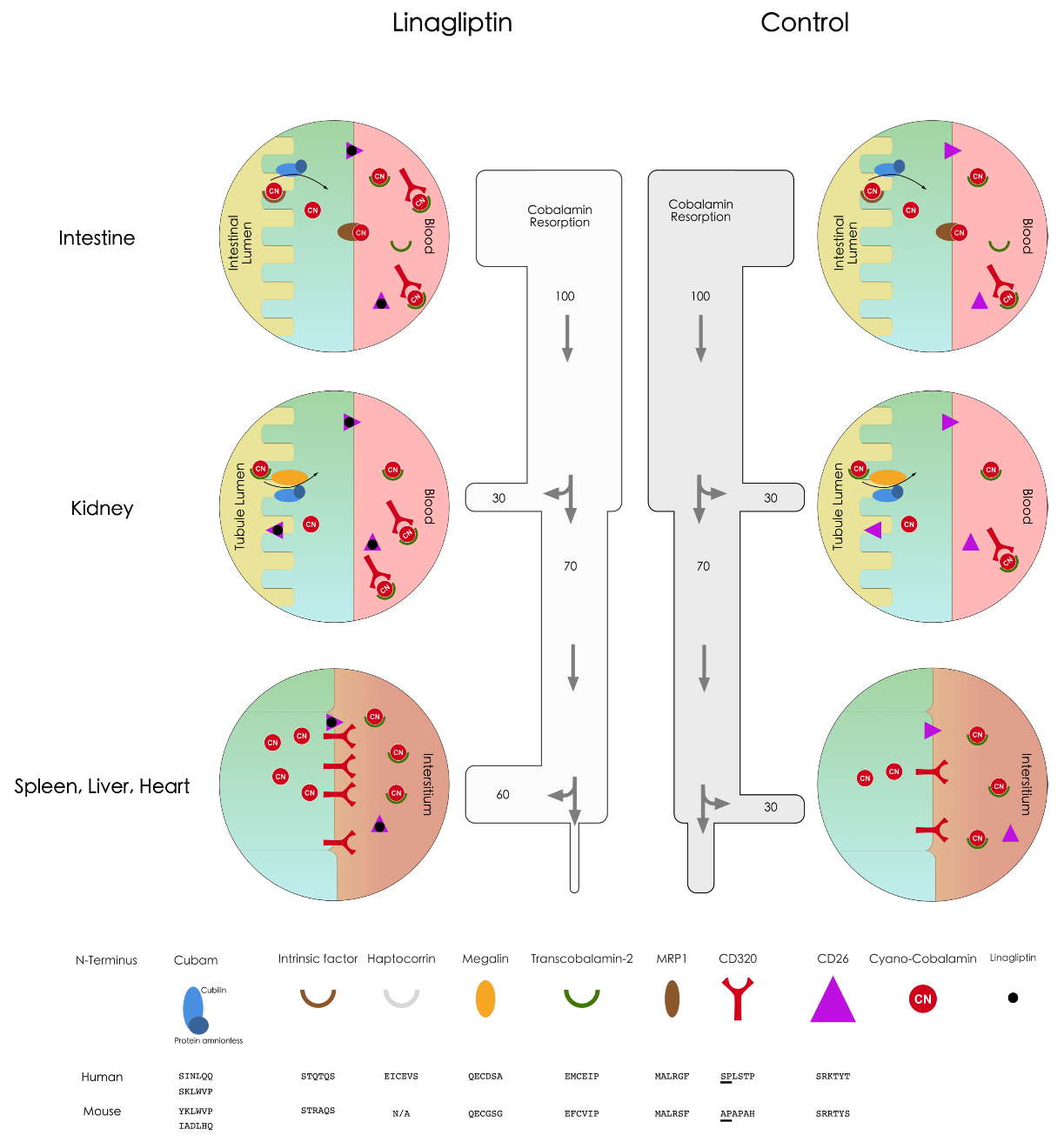
**

Schematic overview of uptake and transport of cobalamin and possible effects of linagliptin treatment.

In the upper gastrointestinal tract, cobalamin is released from food components and is bound by haptocorrin (only in humans and not in mice). On reaching the duodenum, haptocorrin is degraded by enzymes and cobalamin is captured by intrinsic factor secreted by parietal cells of the mucosa of the gastric wall. In the terminal ileum, intrinsic-factor-bound cobalamin is endocytosed by cubam. Inside the ileal enterocyte, intrinsic factor is degraded and cobalamin is released to plasma from the basolateral side of the cell by MRP1. In plasma, cobalamin is bound to haptocorrin or transcobalamin or as TC-Cbl bound to soluble CD320. The latter two complexes are responsible for delivery of cobalamin to cells. In the kidney the receptor megalin mediates renal reabsorption whereas in the liver and other tissues, transcobalamin-dependent cellular cobalamin uptake is mediated by the receptor CD320.

Hypothetically treatment with linagliptin (right) increases CD320 density in organs (spleen, heart, liver) and putatively blood due to CD26 (DPP-4) inhibition and consequently leads to increased intracellular cobalamin. This is indicated by the numerals and shape sizes. The N-terminal six amino acid residues from humans and mice of each protein involved in cobalamin transport are depicted at the bottom and the prototypical DPP-4 consensus cleavage motif is underlined (if present).

DPP-4, dipeptidyl peptidase-4; MRP1, multidrug resistance protein 1; TC-Cbl, transcobalamin-cobalamin.

**Supplementary Figure S5**

**
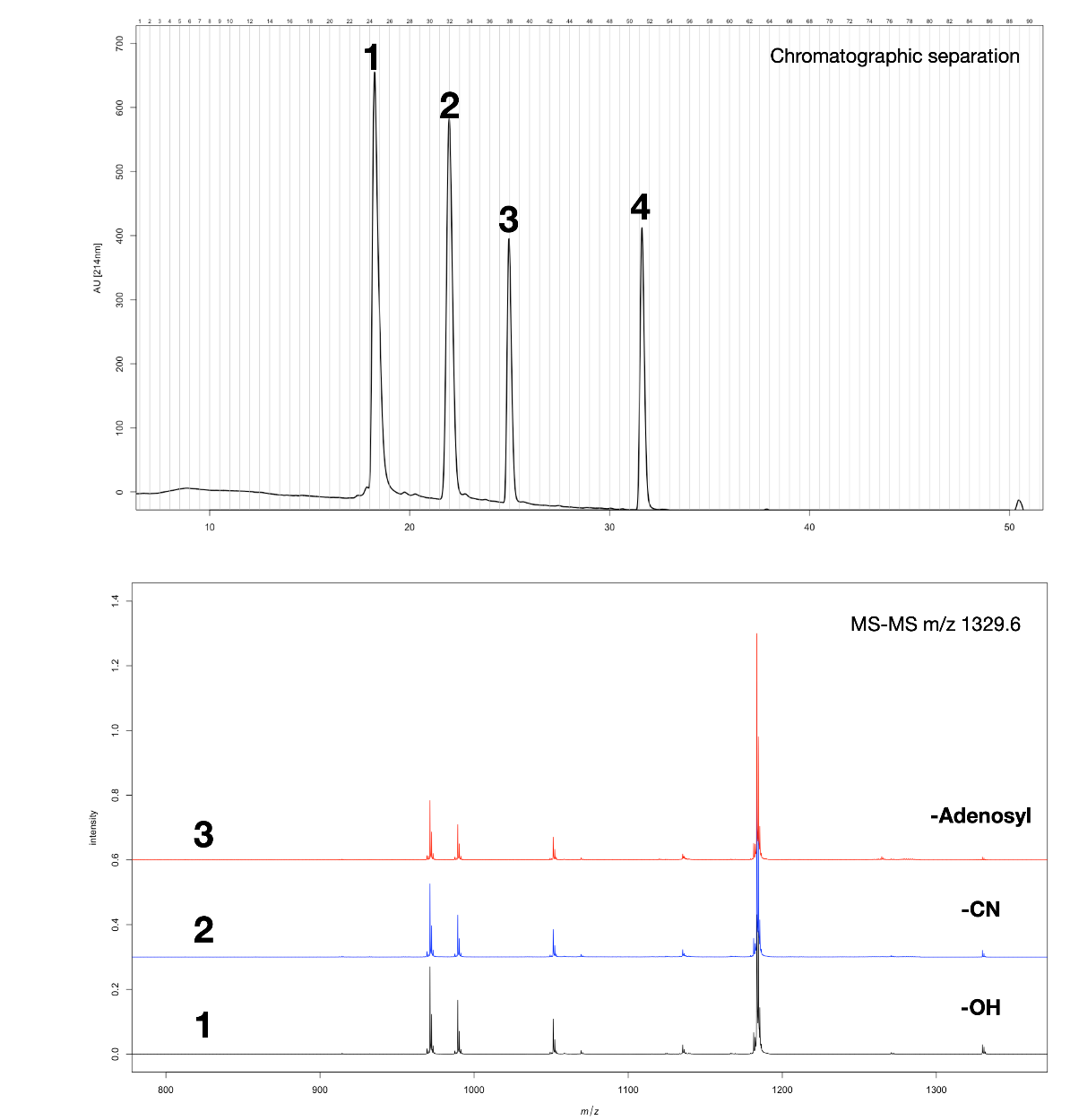
**

Top: Chromatogram of four variants of cobalamin (1: OH, 2: CN; 3: Adenosyl, 4: CH3) after reversed-phase chromatography using an acetonitrile gradient. The y-axis depicts the absorption at 214 nm and the x1-axis the retention time in minutes and the x2-axis collected fractions. Bottom: MS-MS fragmentation spectra of different Cbl derivatives (1: OH, 2: CN; 3: Adenosyl).

Cbl, cobalamin; CH3, methyl; CN, cyano; OH, Hydroxy.
